# Flavonoid Glycosides in *Brassica* Species Respond to UV-B Depending on Exposure Time and Adaptation Time

**DOI:** 10.3390/molecules26020494

**Published:** 2021-01-18

**Authors:** Susanne Neugart, Christiane Bumke-Vogt

**Affiliations:** 1 Division Quality and Sensory of Plant Products, Georg-August-Universität Göttingen, Carl-Sprengel-Weg 1, 37075 Goettingen, Germany; 2 Leibniz-Institute of Vegetable and Ornamental Crops, Theodor-Echtermeyer-Weg 1, 14979 Grossbeeren, Germany; bumke@igzev.de

**Keywords:** quercetin glycosides, kaempferol glycosides, ultraviolet radiation, kaempferol-3-caffeoyl-sophoroside-7-glucoside, sinapic acid

## Abstract

Recently, there have been efforts to use ultraviolet-B radiation (UV-B) as a biotechnological tool in greenhouses. Leafy *Brassica* species are mainly considered for their ability to synthesize glucosinolates and are valued as baby salads. They also have a remarkable concentration of chemically diverse flavonoid glycosides. In this study, the effect of short-term UV-B radiation at the end of the production cycle was investigated without affecting plant growth. The aim was to verify which exposure and adaptation time was suitable and needs to be further investigated to use UV as a biotechnological tool in greenhouse production of *Brassica* species. It is possible to modify the flavonoid glycoside profile of leafy *Brassica* species by increasing compounds that appear to have potentially high antioxidant activity. Exemplarily, the present experiment shows that kaempferol glycosides may be preferred over quercetin glycosides in response to UV-B in *Brassica rapa* ssp. *chinensis*, for example, whereas other species appear to prefer quercetin glycosides over kaempferol glycosides, such as *Brassica oleracea* var. *sabellica* or *Brassica carinata*. However, the response to short-term UV-B treatment is species-specific and conclusions on exposure and adaptation time cannot be unified but must be drawn separately for each species.

## 1. Introduction

While one challenge in today’s agriculture and horticulture is to ensure food security for the world’s growing population, on the other hand, health-promoting foods for an aging population are in high demand [1]. *Brassica* species are known for their content of glucosinolates. However, the remarkable amounts of flavonoids, a well-known group of antioxidants, are rarely considered in *Brassica* species. Flavonoids are ubiquitous in plants [2]. The most important flavonoids in the human diet are quercetin and kaempferol, which are present as complex glycosides in *Brassica* species [3,4,5]. Flavonoids act as antioxidants [6] and shielding components [7] in plants and are of special interest due to their antioxidant activity, as well as anti-inflammatory and anti-cancerogenic effects in humans [8,9,10].

*Brassica* plants are close relatives and include six species described by the U-triangle, three of which are diploid (*Brassica rapa* n = 10 (AA), *Brassica nigra* n = 8 (BB), and *Brassica oleracea* n = 9 (CC)), while the other three are allotetraploid hybrids (*Brassica juncea* n = 18 (AABB), *Brassica napus* n =19 (AACC), *Brassica carinata* n = 17 (BBCC)) derived from each pair of the three diploid species. *Brassica* species that share the same genome are thought to respond similarly to UV-B. The major flavonoids in various *Brassica* species are the complexes kaempferol 3-*O*-sophoroside-7-*O*-glucoside and quercetin 3-*O*-sophoroside-7-*O*-glucoside and their hydroxycinnamic acid acylated forms [4,11,12]. Currently, the consumption of raw leafy *Brassica* species as baby leaf salads gained worldwide attention and provides an opportunity to increase flavonoids in the human diet.

Ultraviolet radiation (UV; 290–400 nm) comprises a relatively minor fraction of the total solar radiation reaching the earth’s surface and is known to play an important role in regulating the growth, photosynthesis, and secondary plant metabolites of higher plants [13,14]. Most plants grown in greenhouses and plastic tunnels lack adaptation to UV radiation during their growth. Consequently, they have lower concentrations of flavonoids [15]. In modern agriculture, UV radiation has been considered harmful to plants because it reduces plant photosynthesis and thus plant growth. In contrast, traditional farming techniques increase sunlight and simultaneously UV radiation during plant growth and ripening to increase secondary plant metabolites that contribute to flavor and color, e.g., reduction of leaves in the vine during berry ripening [16,17]. Recent studies, however, have highlighted the regulatory properties of low, ecologically relevant UVB levels that trigger the accumulation of certain flavonoids, known for their high antioxidant activity, in *Brassica* species [11,18,19,20,21] and other species [14,22]. Flavonoid enrichment also affects plant quality and is important in meeting consumer preferences for fruits and vegetables, including growth and appearance, aroma/smell, flavor, color, and compounds that promote plant and human health [14].

In this study, the effect of UV-B treatment on leafy *Brassica* vegetables is demonstrated by including all six *Brassica* species reflecting the *Brassica* U triangle. The aim was to show the effect of short-term UV-B radiation at the end of the production cycle without affecting plant growth, and to look at the gene expression of enzymes involved in the biosynthesis of flavonoid glycosides and hydroxycinnamic acid derivatives during this period, in order to verify for further studies which exposure time and adaptation period is suitable to use UV as a biotechnological tool in greenhouse production for different *Brassica* species.

## 2. Results

**Figure 1:**
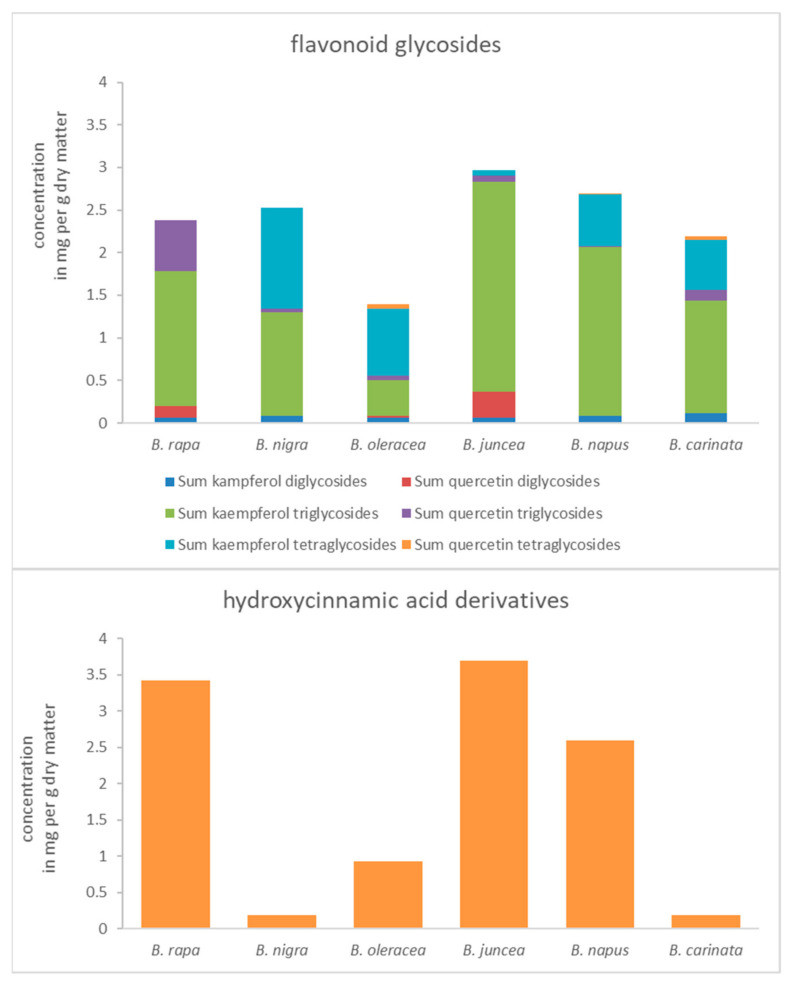
Concentration of flavonoid glycoside groups (separated on the type of aglycone and the glycosylation pattern) and hydroxycinnamic acid derivatives of related *Brassica* species (mean of all control plants) in mg per g dry matter.

**Figure 2:**
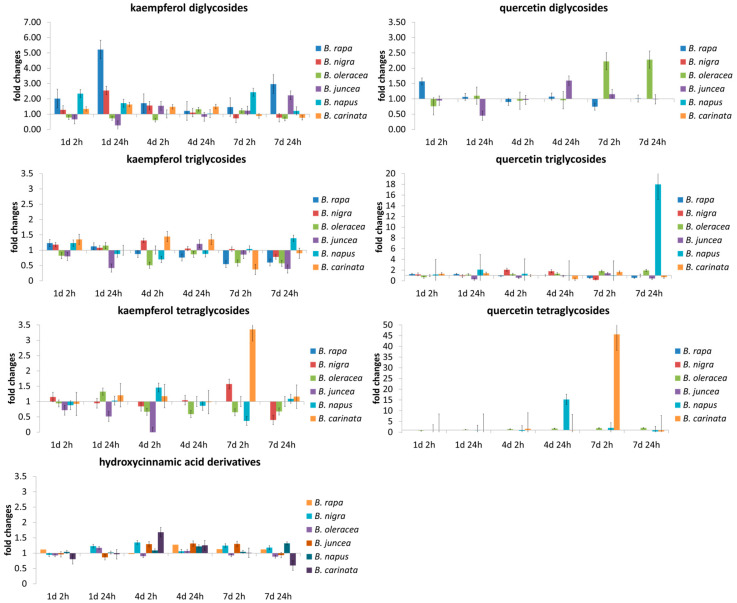
Relative changes in the concentration of flavonoid glycoside groups (separated on the type of aglycone and the glycosylation pattern) and hydroxycinnamic acid derivatives of related *Brassica* species normalized on the value of the control plants at the certain time points (at days 1, 4, and 7 of the treatment with 2 h or 24 h of adaptation time after the last UV treatment).

**Figure 3:**
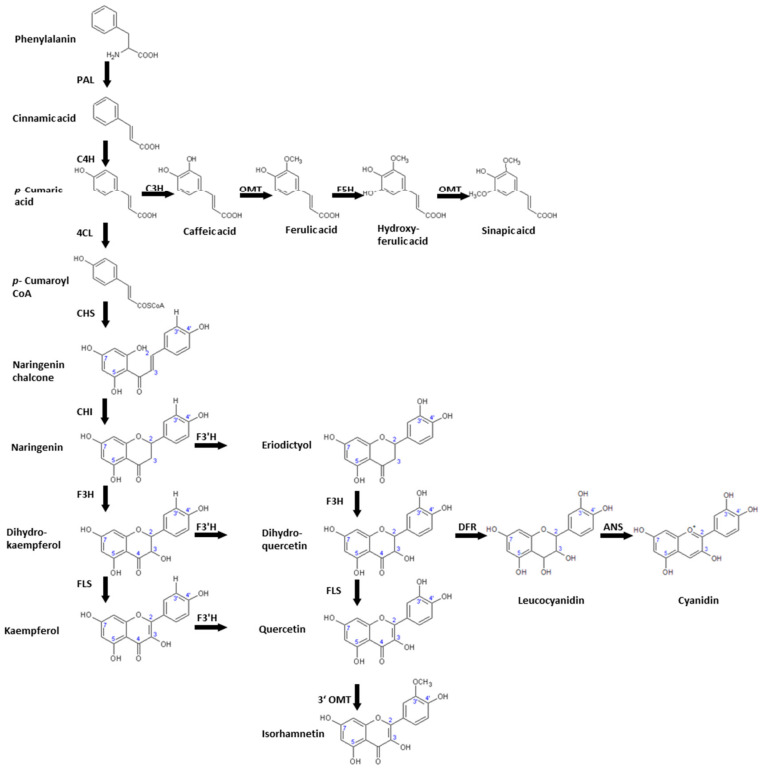
Biosynthesis of phenylpropanoids relevant to *Brassica* species including the involved enzymes PAL: phenylalanine ammonia-lyase, C4H: cinnamic acid 4-hydroxylase, C3H: p-coumaric acid 3-hydroxylase, OMT: *O*-methyltransferase, F5H: ferulic acid 5-hydroxylase, 4CL: 4-Coumaroyl CoA-Ligase CHS: chalcone synthase, CHI: chalcone isomerase, F3H: flavanone 3β-hydroxylase, FLS: flavonol synthase, F3′H: flavonol 3′-hydroxylase, 3′ OMT: 3′-*O*-methyltransferase, DFR: dihydroflavonol 4-reductase, ANS: anthocyanin synthase.

**Figure 4:**
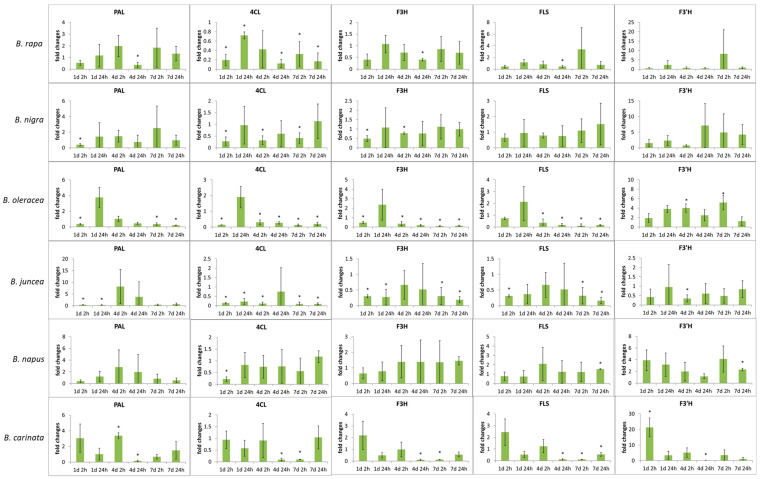
Relative expression of genes presumably coding for phenylalanine ammonia-lyase (*PAL*), 4-Coumaroyl CoA-Ligase (*4CL*), flavanone 3β-hydroxylase (*F3H*), flavonol synthase *(FLS*), flavonol 3′-hydroxylase (*F3′H*) normalized on the basis of expression of actin at the certain time points (at days 1, 4, and 7 of the treatment with 2 h or 24 h of adaptation time after the last UV treatment). Significant differences are highlighted by an asterix at *p* ≤ 0.05.

Six leafy *Brassica* species representing the *Brassica* U triangle were analyzed for their flavonoid glycoside accumulation (Figure 1) after short-term UV-B treatment (Tables S1–S6) at the end of their production cycle as baby leaf salads. In addition, hydroxycinnamic acid derivatives (Figure 1; Table S7) were studied. The effect of UV-B treatment on flavonoid groups and hydroxycinnamic acid derivatives was studied for each *Brassica* species (Figure 2). Accompanying the metabolites, gene expression of key genes in the phenylpropanoid pathway (Figure 3) was analyzed to specify the role of specific enzymes in the response to UV-B (Figure 4).

### 2.1. Flavonoid Glycosides and Hydroxycinnamic Acid Derivatives in Related Brassica Species

All studied *Brassica* species (Figure 1) showed high concentrations of acylated kaempferol triglycosides, while some of them also contain remarkable amounts of kaempferol tetraglycosides.

The concentration of quercetin tri- and tetraglycosides was lower compared to the kaempferol tri- and tetraglycosides. However, in the case of diglycosides, which are a minor fraction of flavonoid glycosides in *Brassica*, the concentrations of quercetin glycosides were higher than those of kaempferol glycosides in *B. rapa* ssp. *chinensis* (genome AA) and *B. juncea* (genome AABB). In detail, *B. rapa* ssp. *chinensis* was mainly characterized by acylated kaempferol di- and triglycosides, whereas kaempferol or quercetin tetraglycosides were absent. In addition to acylated kaempferol di- and triglycosides, *B. nigra* (genome BB) contained acylated kaempferol tetraglycosides, whereas corresponding quercetin glycosides were generally absent at higher concentrations. In contrast to the two previously mentioned, *B. oleracea* var. *sabellica* (genome CC) contained acylated kaempferol and quercetin di-, tri-, and tetraglycosides, representing the highest diversity of flavonoid glycosides among the *Brassica* species studied. However, the concentration of flavonoid glycosides in *B. oleracea* var. *sabellica* is low compared to the other *Brassica* species studied. Interestingly, *B. juncea* has higher concentrations of quercetin diglycosides but lower concentrations of quaercetin triglycosides than *B. rapa* ssp. *chinensis*. In general, it contained the highest concentrations of flavonoid glycosides. On the other hand, *B. napus* (genome AACC) has a lower number of quercetin glycosides while it contains a diversity of kaempferol di-, tri-, and tetraglycosides, clearly showing the influence of the *B. oleracea* var. *sabellica*. The last species *B. carinata* (genome BBCC) is characterized by a high diversity of flavonoid glycosides including kaempferol and quercetin di-, tri- and tetraglycosides and therefore is comparable to *B. oleracea* var. *sabellica* regarding the flavonoid glycoside profile.

Hydroxycinnamic acid derivatives (Figure 1) were highly concentrated in *B. rapa* ssp. *chinensis* and *B. juncea* and *B. napus*, but low concentrated in the other species not containing the AA genome.

### 2.2. The Effect of UV-B, Exposure Time, and Adaptation Time on Flavonoid Glycosides and Hydroxycinnamic Acid Derivatives in Related Brassica Species

The concentrations of flavonoid glycosides and hydroxycinnamic acid derivatives increased with plant development over the 7 days of the experiment. For most of the species studied, the effect of the UV-B treatment was below 50% increase or decrease, with some exceptions that depended on species and exposure or adaptation time (Figure 2). While kaempferol diglycosides were involved in the early response to UV-B, kaempferol tri- and tetraglycosides were less intensely altered by UV-B in all *Brassica* species. In contrast, in *B. oleracea* var. *sabellica* quercetin glycosides generally appear to be the line of defense against UV-B with a dose–response relationship (Figure 2). In *B. nigra*, *B. napus*, and *B. carinata* quercetin tri- and tetralycosides are important for UV-B response on later days. In the results of ANOVA (Tables S1–S7) and subsequent Tukey’s HSD (data not shown), mention is made only when both resulted in a significant change.

Kaempferol diglycosides (Table S1) show a species specific response to short-term UV-B among the *Brassica* species studied. While in *B. rapa* ssp. *chinensis*, caffeic acid acylated kaempferol diglycoside was increased after 24 h of adaptation on day 1, ferulic acid acylated kaempferol diglycoside was increased on day 7 after 2 and 24 h of adaptation. In *B. nigra*, caffeic acid acylated and hydroxyferulic acid acylated kaempferol diglycosides were increased day 1. Later, no effect of UV-B was found, except for an increase in sinapic acid acylated kaempferol diglycoside after 2 h of adaptation on day 4. At the same time, in *B. oleracea* var. *sabellica*, *B. juncea*, and *B. napus*, hardly any effects of UV-B on kaempferol diglycosides are detectable, whereas in *B. carinata* hydroxyferulic acid acylated kaempferol diglycoside increased after 2 h of adaptation on day 1 and sinapic acid acylated kaempferol diglycoside after 24 h of adaptation on day 1. Quercetin diglycosides (Table S2) were not affected by the UV-B treatment regardless of exposure time and adaptation time, except for quercetin-3-sophoroside, which was increased after 2 h of adaptation on day 7 in *B. oleracea* var. *sabellica*.

The triglycosides of kaempferol and quercetin (Tables S3 and S4) were affected by short-term UV-B treatment not only by species but also as a function chemical structural characteristics including the aglycone and the acylated hydroxycinnamic acid. In *B. rapa* ssp. *chinensis*, *B. nigra*, and *B. napus* only a small number of kaempferol and quercetin triglycosides were induced at all by the UV-B treatment. However, in *B. rapa* ssp. *chinensis*, the ferulic acid acylated kaempferol triglycoside was decreased at day 7 after 2 h adaptation. Changes were observed in *B. nigra* towards the beginning of the treatment including the increase of hydroxyferulic acid acylated quercetin triglycoside after 2 h of adaptation of day 1 followed by the decrease of the same compound after 24 h of adaptation at day 1. In contrast to the two before mentioned species, *B. oleracea* var. *sabellica* showed a high response of kaempferol and quercetin triglycosides starting after 2 h of adaptation on day 4 and established on day 7 as evidenced mainly by the decrease of non-acylated and acylated kaempferol glycosides and an increase of coumaric acid acylated kaempferol triglycoside and caffeic acid acylated quercetin triglycoside on day 4 after 2 and 24 h of adaptation, respectively. The hybrids *B. juncea* and *B. napus* showed a decrease in triglycosides or no effects of the UV-B treatment on the kaempferol and quercetin triglycosides, whereas *B. carinata* showed a high response of kaempferol and quercetin triglycosides in the same way as *B. oleracea* var. *sabellica*. Interestingly, in *B. carinata*, non-acylated and acylated kaempferol increased at day 4 mainly at 2 h of adaptation and quercetin-3-hydroxyferuloyl-sophoroside-7-glucoside was increased at day 1 regardless of adaptation time, but no effect was found at day 7.

No kaempferol or quercetin tetraglycosides (Tables S5 and S6) were detected in *B. rapa* ssp. *chinensis*. No effects were detected for the kaempferol tetraglycosides in *B. nigra* and the hybrids *B. juncea* and *B. napus*. However, the tetraglycosides of *B. oleracea* var. *sabellica* responded strongly to the short-term UV-B treatment. Most kaempferol tetraglycosides were decreased at day 7 independent of the adaptation time, except the hydroxyferulic acid acylated kaempferol tetraglycoside, which was increased after 2 and 24 h adaptation on day 7. A higher number of tetraglycosides were identified in *B. carinata*, of which the sinapic acid acylated kaempferol tetraglycoside increased after 24 h adaptation on day 4, whereas the disinapic acid acylated kaempferol tetraglycoside decreased after 24 h of adaptation on day 7. Quercetin tetraglycosides were not affected by UV-B, with the exception of quercetin-3-sinapoyl-sohoroside-7-diglucoside, which was increased in *B. oleracea* var. *sabellica* at days 4 and 7 regardless of the adaptation time.

The effect of UV-B on hydroxycinnamic acid derivatives (Table S7) was investigated. *B. nigra*, *B. oleracea* var. *sabellica*, and *B. carinata* contain the typical hydroxycinnamic acid derivatives chlorogenic acid, disinapoyl-gentionbioside and sinapoyl, feruloyl-gentiobioside, trisinapoyl-gentiobioside and disinapoyl, feruloyl-gentiobioside. In *B. nigra*, only cholorogenic acid was used in early UV-B defense, resulting in an increase on day 1 after 2 h of adaptation. *B. oleracea* var. *sabellica* trisinapoyl-gentiobiose was increased on day 4 and day 7 after 2 h of adaptation, while disinapoyl, feruloyl-gentiobiose was increased on day 4 after 2 h of adaptation only. In agreement with this, in B. carinata disinapoyl-gentibioside and trisinapoyl-gentiobioside were increased on day 4 regardless of the adaptation time and the latter one was also increased on day 7 at 24 h of adaptation. At the same time, cholorogenic acid was decreased after 2 h of adaptation on day 7. *B. rapa* ssp. *chinensis*, *B. juncea*, and *B. napus* were able to synthesize hydroxycinnamoly malates and preferentially used them in the UV-B response. In *B. rapa* ssp. *chinensis*, cholorgenic acid decreased after 2 h of adaptation on day 4 and hydroxyferuloyl malat decreased after 24 h of adaptation on the same day, whereas in *B. juncea* coumaroyl malate, feruloyl malate, and sinapoyl malate increased after 24 h of adaptation on day 4. In parallel, in *B. napus* coumaroyl malate and sinapoyl malate increased after 24 h of adaption on day 4, with sinapoyl malate already enhanced after 2 h of adaptation.

### 2.3. The Effect of UV-B, Exposure Time and Adaptation Time on Gene Expression in Related Brassica Species

In all *Brassica* species, the gene expression of enzymes (Figure 3) included in flavonoids synthesis tended to be reduced or unaffected by UV-B treatment, reflecting marginal changes in flavonoid glycosides (Figure 4).

In *B. rapa* ssp. *chinensis*, a downregulation of *PAL* gene as key enzyme of flavonoid synthesis was found 24 h after UV-B treatment on day 4. Comparably, in *B. carinata*, an induction of PAL was observed 2 h after UV-B treatment on day 4 as early reaction that was downregulated after 24 h. Most *Brassica* species show a decrease of *4CL* gene right at the beginning on day 1 after 2 h of adaptation. In *B. rapa* ssp. *chinensis*, *B. oleracea* var. *sabellica* and *B. juncea* a constant reduction of *4CL* gene was found with some exceptions. Similarly, the expressions of *F3H* and *FLS* genes were reduced in *B. oleracea* var. *sabellica* and *B. juncea* but not in *B. rapa* ssp. *chinensis*. *F3′H* gene mRNA expression was downregulated after adaptation to UV-B on day 4 after 24 h in *B. carinata* after early induction at day 1 after 2 h, whereas in *B. oleracea* var. *sabellica* and *B. napus F3′H* gene was induced at day 7 after 2 h and 24 h, respectively. In both species, quercetin glycosides were simultaneously increased at the corresponding time points (Figure 4).

## 3. Discussion

Leafy *Brassica* species are a rich source of structurally different flavonoid glycosides, especially high complex kaempferol and quercetin tri- and tetraglycosides. This is generally confirmed in the literature for *B. rapa* ssp. *chinensis* [3], *B. nigra* [23], *B. oleracea* var. *sabellica* [24,25,26,27], *B. juncea* [28,29], *B. napus* [27,30,31,32], and *B. carinata* [12]. Therefore, they are suitable for human consumption and recently gained attention as raw salads.

### 3.1. The Effect of UV-B on Flavonoid Glycosides in Related Brassica Species

Flavonoids are known to be enhanced by UV-B radiation as a part of the plant’s defense and by aging of the plant [33]. Here B-ring poly-hydroxylated compounds such as quercetin are favored in the UV-B response over B-ring mono-hydroxylated compounds such as kaempferol [34]. The results shown here, in contrast, underline previous results on *B. oleracea* var. *sabellica* [20,35] that the response to UV-B is dependent on the chemical structure of the compound and the duration of the UV-B treatment as well as the adaptation time. It can be concluded that kaempferol glycosides also contribute to the UV-B response, which was previously reported for *B. rapa* ssp. *chinensis* [18], *B. oleracea* var. *sabellica* [20] and *B. napus* [11]. However, the present results were conducted under 2-6 fold higher PAR conditions and also higher temperature than the previous study on *B. oleracea* var. *sabellica* [20], which clearly had an effect on the flavonoid biosynthesis and led to remarkably lower concentrations of quercetin and kaempferol glycosides in the present experiment. The present experiment shows that quercetin glycosides are not always preferred over kaempferol glycosides, as exemplified for *B. rapa* ssp. *chinensis*, where UV-B treatment generally had a minor effect. However, some species seem to prefer quercetin glycosides over kaempferol glycosides, such as *B. oleracea* var. *sabellica* or *B. carinata*, both of which showed similar response to UV-B and carry the CC genome. This has already been shown for *B. oleracea* var. *sabellica* in previous studies [20]. In the present study, *B. napus* did not show high concentrations of quercetin glycosides and did not induce the concentrations of quercetin glycosides due to the UV-B treatment, which is in contrast to the results of Olsson et al. 1998 [11], who used much higher doses of UV-B in the greenhouse. A catechol structure is also found in the acylated hydroxycinnamic acid of the flavonoid glycoside. While caffeic acid and hydroxyferulic acid have a catechol structure, coumaric acid, ferulic acid, and sinapic acid do not. This does not necessarily reflect their ability as antioxidants [36]. The antioxidant activity of compounds including sinapic acid may be remarkable [36]. This is supported by the fact that most *Brassica* species, including *B. nigra*, *B. oleracea* var. *sabellica*, *B. juncea,* and *B. carinata* also increase sinapic acid acylated kaempferol or quercetin glycosides in response to UV-B while *B. rapa* ssp. *chinensis* and *B. napus,* both of which carry the AA genome, do not. However, Harbaum-Piayda et al. 2010 [18] showed an increase in non-acylated and coumaric acid acylated kaempferol triglycosides in *B. rapa* ssp. *chinensis* at 22 °C after 7 days of UV-B treatment, but with higher doses of UV-B at very low PAR. In general, it is known that the intensity of the UV-B doses can be percieved by plants [37] and that the UV/PAR ratio also affects the efficiency of the UV-B exposure [38,39]. In the present experiment, the UV-B dose was low and the PAR was ambient sunlight, which is relatively high. This is one of the challenges transferring the results on UV-B obtained from climate chamber experiments to greenhouses for use as biotechnological tool. While *B. oleracea* var. *sabellica*, *B. juncea,* and *B. carinata* show a higher response of kaempferol glycosides acylated with a hydroxycinnamic acid including a catechol structure, this was not the case for the other *Brassica* species. The catechol structure not only in the flavonoid aglycone but also in the acylated hydroxycinnamic acid is associated with higher antioxidant activity, but there might be interactions within the molecule [36].

### 3.2. The Effect of UV-B on Hydroxycinnamic Acid Derivatives in Related Brassica Species

Hydroxycinnamic acid derivatives from *Brassica* species [40] have rarely been studied as UV-B protectants. The representative hydroxycinnamic acids of *Brassica* are chlorogenic acid, disinapoyl-gentionbioside and sinapoyl, feruloyl-gentiobioside, trisinapoyl-gentiobioside, and disinapoyl, feruloyl-gentiobioside [3,4,25]. In addition to those previously mentioned, *B. rapa* ssp. *chinensis*, *B. juncea,* and *B. napus*, all of which contain the genome AA, are capable of synthesizing malates of hydroxycinnamic acids [3,28,30]. In the present study, the hydroxycinnamic acid malates of *B. rapa* ssp. *chinensis* were not intensively used for UV-B response, which is consistent with results from Harbaum-Piayda et al. 2010 [18], who found no effect of UV-B at 22 °C in *B. rapa* ssp. *chinensis*. However, these compounds seem to play a crucial role in the UV-B response of *B. juncea* and *B. napus*. In *B. nigra*, *B. oleracea* var. *sabellica,* and *B. carinata* chlorogenic acid and the sinapic acid acylated glycosides take over the response to UV-B, although no effects of UV-B treatment on these compounds were previously described in *B. oleracea* var. *sabellica* [20].

### 3.3. The Effect Exposure Time and Adaptation Time on Flavonoid Glycosides and Hydroxycinnamic Acid Derivatives in Related Brassica Species

The species also differ in their temporal response. While *B. rapa* ssp. *chinensis*, *B. nigra*, *B. napus,* and *B. carinata* show an immediate positive response and *B. juncea* shows a negative response starting on day 1 but lasting only until day 4, *B. oleracea* var. *sabellica* has a delayed response starting on day 4 and continuing until day 7. The duration of the UV-B treatment is one of the major challenges in implementing this technique into greenhouses [14]. While some studies show an adaptation of plants within h [41,42] other studies indicate that constant UV-B treatment over a longer period is required [43,44,45,46]. In particular, an increase in flavonoids after several UV-B treatments over several days has been reported in the literature, as shown for *Arabidopsis thaliana*, a model plant and relative of *Brassica* [47], such as *Brassica oleracea* var. *sabellica* [20]. An increase in sinapoyl, feruloyl-gentiobiose after 24 h adaptation as well as trisinapoyl-gentiobiose and disinapoyl, feruloyl-gentiobiose at day 4 was found in the present study for *B. oleracea* var. *sabellica* and previously an increase in these compounds in response to UV-B at high PAR was described for *B. oleracea* var. *italica* [48]. The present study highlights that in *Brassica* species, the effect of UV-B is highly dependent on the species itself. Therefore, tailored UV radiation recipes need to be developed separately for each species, especially when a UV-B treatment is considered at the end of the production cycle. A second aspect was investigated in the present study, which is the adaptation time. In *A. thaliana*, it was shown that a higher number of genes were affected by high light treatment after 24 h then after 2 h [49]. Higher light treatment was also associated with higher concentrations of kaempferol glycosides in *A. thaliana* [50] The present study suggests that there are fast responding species that increase their quercetin glycosides within 2 h of adaptation, such as *B. oleracea* var. *sabellica* and *B. carinata*, while in others biosynthesis is slower, resulting in changes after 24 h, e.g., in the hybrid *B. napus*.

### 3.4. The Effect of UV-B, Exposure Time, and Adaptation Time on Gene Expression in Related Brassica Species

In general, the gene expression results represent the findings for flavonoid glycosides. In some species such as *B. juncea* and *B. napus* gene expression was comparable across the exposure time and adaptation time, highlighting the small effect the UV-B treatment had on these plants. On the other hand, species such as *B. rapa* var. *chinensis*, *B. oleracea* var. *sabellica* and *B. carinata* showed that gene expression was altered over both the exposure time as well as the adaptation time. This demonstrates how rapidly and specifically some *Brassica* species can adapt their flavonoid profile and concentration as needed. Although downstream gene expression starting at PAL and including 4CL, F3H, and F3′H has been proposed in *A. thaliana* [51], present data highlight that specific gene expression occurs in response to UV-B by increasing F3′H specifically in *B. oleracea* var. *sabellica*, *B. napus,* and *B. carinata*. The complexity of gene expression in relation to flavonoid biosynthesis was previously shown in *A. thaliana* by combining intrinsic and extrinsic factors [52]. The importance of flavonols such as quercetin and kaempferol in the absorption of UV and blue light was shown in *A. thaliana* mutants (tt5-CHI) and to their absence resulted in higher damage in the chloroplast [53]. However, the expression of PAL, 4CL, F3H, and FLS was mostly downregulated in the *Brassica* species, which may be a feedback to the high F3′H gene expression or a consequence of the high concentrations of kaempferol glycosides already present in *Brassica* species, sufficient for the UV response. However, F3′H gene expression was increased in *B. oleracea* var. *sabellica* and *B*. *carinata*, which simultaneously resulted in higher concentrations of quercetin glycosides, whereas it was hardly affected in other *Brassica* species. This supports the results of the metabolite analysis showing that *B. oleracea* var. *sabellica* and *B. carinata* favor quercetin quercetin glycosides in the response to UV-B, while the other *Brassica* species do not. The decrease of 4CL gene expression did not necessarily lead to higher concentrations of hydroxycinnamic acid derivatives, as exemplified in *B. oleracea* var. *sabellica*. While *B. rapa* var. *chinensis* is a species grown in the subtropics and tropics, *B. oleracea* var. *sabellica* is an established vegetable grown in winter in Central Europe. These different growing conditions may have influenced the sesitivity to reactive oxygen species and the ability of the plant to synthesize flavonoid glycosides and hydroxycinnamic acid derivatives as defense compounds.

## 4. Materials and Methods

### 4.1. Plant Material and Experimental Design

Seeds of *Brassica rapa* ssp. *chinensis* (pak choi) cv Black Behi (By Allied Botanical, Quezon City, Philippines), *Brassica oleracea* var. *sabellica* (kale) cv Winterbor (by Bruno Nebelung, Norken, Germany), *Brassica juncea* cv Mizuna, *Brassica napus* und *Brassica nigra* (all by Albert Treppens & Co Samen GmbH, Berlin, Germany), and *Brassica carinata* (by the World Vegetable Center (AVRDC) Arusha, Tanzania) were sown on 9 September 2016 in potting soil (Einheitserde Type 1, Fitz Kausek GmbH & Co.KG, Mittenwalde, Germany) and set in a randomized block. Water was supplied as required by the plants and fertilizer was administered every two weeks as a nutrient solution (pH 6.4, P 41 mg L^−1^, Mg 43 mg L^−1^, NO_3_ 154 mg L^−1^, Ca 205 mg L^−1^, K 230 mg L^−1^, SO_4_ 280 mg L^−1^, Zn 0.82 mg L^−1^). All plants were grown to the 4–5 leaf stage under greenhouse conditions (temperature 20 °C and PAR mean 603 µmol m^−2^s^−1^ (82–1167 µmol m^−2^s^−1^)) in Grossbeeren (Germany, 52.37° N 13.33° E) under partly cloudy skies. Plants were exposed to up to seven consecutive daily doses of 0.5 kJ m^−2^ d^−1^ UV-B radiation (1 h UV-B (10:00 to 11:00) with acclimation intervals of 23 h), which corresponds to a daily dose of one winter day in central Europe [54] and is thus moderate among the PAR doses used. This daily dose was previously applied to greenhouse-grown kale and resulted in an increase in quercetin glycosides after 3 or more days of exposure [20]. UV-B radiation was supplied by five UV-B fluorescent light sources (TL 40W 12 RS, Philips, Hamburg, Germany) and the distance of the UV-B lamps to the plants was 80 cm. UV-B radiation was determined using a UV-B sensor (type DK-UVB 1.3–051, deka Sensor + Technologie, Teltow, Germany) with a spectral range of 265 to 315 nm. The corresponding biologically effective UV-B irradiance of 0.25 kJ m^−2^ d^−1^ UV-B_BE_ was calculated using Caldwell’s generalized plant weighting function. Two plants per replicate and three biological replicates per treatment were harvested from the plant block on days 1, 4, and 7 after an acclimation period of 2 h or 24 h after UV-B treatment.

### 4.2. Sample Preparation

Leaves (without midrib) were collected from three plants at the 4- to 5-leaf stage and frozen in liquid nitrogen. One aliquot was stored at −80 °C for gene expression analysis, and one aliquot was stored at −40 °C until lyophilization, lyophilized, and then ground to a powder. Samples were stored in the dark at room temperature until needed for analysis. For analysis of flavonol glycosides, samples were extracted as previously described [12].

### 4.3. HPLC-DAD-ESI-MS^n^

The HPLC-DAD-ESI-MS^n^ method for the tentative identification and quantitation of the flavonol glycosides in *Brassica* species was done with an HPLC series 1100 by Agilent (Waldbronn, Germany) consisting of a degaser, binary pump, autosampler, column oven, and photodiode array detector was used. An ion trap (Agilent series 1100 MSD) with an ESI ion source in negative ionization mode was used as the mass spectrometer. The parameters for the HPLC-DAD-ESI-MS^n^ were formerly described [35]. Mass fragments for the identification of the compounds were previously given [26]. The standards quercetin-3-*O*-glucoside (for quercetin glycosides) and kaempferol-3-*O*-glucoside (for kaempferol glycosides) were used to obtain an external calibration curve ranging from 0.1 to 10 mg 100 mL^−1^ for a semi-quantitative approach.

### 4.4. Gene Expression Analysis by Semi-Quantitative Real-Time PCR

RNA was extracted from an aliquot (10 mg) of the identical plant material as used for the metabolite analysis using the Nucleo-Spin Plant Kit (Macherey-Nagel GmbH and Co. KG, Dueren Germany), including on-column DNase digestion. RNA was quantified spectrophotometrically at 260 nm (NanoDrop ND1000, NanoDrop Technology Inc., Wilmington, DE, USA); quality was checked using the A 260/A 280 ratio calculation, whereby a ratio between 1.9 and 2.1 was considered acceptable. Single-stranded cDNA synthesis was carried out with total RNA using SuperScript_ III RNaseHreverse transcriptase (Invitrogen GmbH, Darmstadt, Germany) with oligo d(T12-18) primers according to the manufacturer’s instructions. Quantitative two-step RT-PCR was performed using an SYBR Green^®^ protocol in 96-well reaction plates on an Applied Biosystems 7500 Real-time PCR System. The following thermal profile was used for all reactions: 50 °C for 2 min, 95 °C for 10 min, 40 cycles of 95 °C for 30 s, and 60 °C for 1 min, followed by dsDNA melting curve analysis to ensure amplicon specificity. Each reaction was performed in a 10 µL volume containing 200 nM of each primer, 3 µL of cDNA (1:50), and 7 µL of Power SYBR Green Master Mix (Applied Biosystems, Foster City, CA, USA). The gene-specific primer sets are listed in Table 1.

Design of primer pairs for amplification of mRNA regions including one exon-junction was performed using NCBI reference sequences predicted for *Brassica oleracea* flavonoid synthesizing enzymes: XM_013749252.1 for PAL phenylalanin ammonia lyse 2 (LOC106311920), XM_01373510.1 for 4CL 4-coumarate-CoA ligase 3 (LOC106296384), XM_013778875.1 for ANS anthocyanidin synthase (LOC106339986), XM_013753582.1 for DFR Dihydroflavonol-4-reductase or leucoanthocyanidin dioxygenase LDOX (LOC106315758), XM_013758739.1 for FLS3 flavonol synthase 3-like (LOC 106320382), XM_013751217.1 for F3H flavanone 3-hydroxylase (LOC 106313418), XM_013751545.1 for F3′H flavonoid 3′-monooxygenase (LOC106313667), and XM_013753106.1 for housekeeping gene actin-2 (LOC 106315376). Data generated by real-time PCR were collected and compiled using 7500 v2.0.1 software (Applied Biosystems). Data were exported to LinReg software [54] to determine the PCR amplification efficiency for each primer pair. Relative transcript levels were normalized on the basis of expression of an invariant control orthologous to actin-2 using the equation 2^−ΔΔCT^ [55]. Quantitative PCR was performed on three biological replicates measured in duplicates for each gene; non-template controls were included.

### 4.5. Statistical Analysis

All data were statistically analyzed with two-way ANOVA followed by Tukey’s HSD test for selected compounds. Residuals were tested for Gaussian distribution using the Kolmogorov–Smirnov test. All tests were performed at a 5% significance level. Calculations were performed using StatisticaTM for WindowsTM (version 9.0, Statsoft Inc., Tulsa, OK, USA).

## 5. Conclusions

Leafy *Brassica* species, especially *B. oleracea* var. *sabellica* and *B. carinata,* are a suitable target for the implementation of UV-B radiation into greenhouses. It is possible to modify their flavonoid glycoside and hydroxycinnamic acid derivative profile by increasing compounds that seem to have potentially high antioxidant activity. However, the response to a short-term UV-B treatment is species specific and conclusions on exposure time and adaptation time need to be drawn separately for each species. Nevertheless, species sharing the same genome are able to synthesize the same compounds and showed similar responses in some cases. For *B. oleracea* var. *sabellica* and *B. carinata*, the exposure time should be 4 days or more, whereas the adaptation time seems to be different between compounds without clear direction. Other environmental factors, especially PAR present during greenhouse production, should also be included to study the interactions.

## Figures and Tables

**Table 1:** Oligonucleotide primers for genes of the phenolprpanoid biosynthesis and the reference gene based on the genome of *Brassica oleracea* for gene expression analysis by semiquantitative real-time PCR.

Gene Function (Gene Name)	Oligonucleotide Abbreviation	Sequence
Actin (*ACT*)	Bo_ACT2_up1_E4	GCA-CCT-CCC-GAG-AGG-AAG-TA
	Bo_ACT2_lo1_E4/5	CCT-TGG-AGA-TCC-ACA-TCT-GCT
Phenylalanine ammonium lyase (*PAL*)	Bo-up 1 PAL E1	TGG-GTT-ATG-GAA-AGT-ATG-GGC-A
	Bo-lo 1 PAL E1-2	CCG-GCG-TTC-AAA-AAT-CTG-ATG-A
4-coumaroyl CoA ligase (*4CL*)	Bo-up_4CL_E3/4	CCT-TGG-CCA-GGG-ATA-TGG-TA
	Bo-lo_4CL_E4	AGC-TCT-GCG-TTT-CGG-ACT-AC
Flavanone 3β-Hydroxylase (*F3H*)	Bo_F3H_up1_E2/3	TCC-ACC-TGA-GTA-CAG-AGA-GGT
	Bo_F3H_lo1_E3	TTC-TCT-CAA-CGC-CTC-ACG-AC
Flavonol synthase (*FLS*)	Bo-up 6 FLS E2-3	CGG-CGA-TCA-GAT-ACT-GAG-GCT
	Bo-lo 6 FLS E3	TGT-GGC-TCC-AAG-AAC-ACT-GG
Flavonol 3′-hydroxylase (*F3′H*)	Bo¬_F3′H_up2_E3/4	CCG-TAC-CTT-CAG-GCG-GTT-AT
	Bo¬_F3′H_lo2_E4	AGC-CGT-TGA-TCT-CAC-AGC-TC

## Data Availability

The data presented in this study are openly available at https://data.goettingen-research-online.de/dataset.xhtml?persistentId=doi:10.25625/JBWKIL.

## Supplementary Materials

The following are available online, Table S1: Kaempferol diglycosides of different Brassica species in µg/g DW., Table S2: Quercetin diglycosides of different Brassica species in µg/g DW., Table S3: Kaempferol triglycosides of different Brassica species in µg/g DW., Table S4: Quercetin triglycosides of different Brassica species in µg/g DW., Table S5: Kaempferol tetraglycosides of different Brassica species in µg/g DW., Table S6: Quercetin tetraglycosides of different Brassica species in µg/g DW., Table S7: Quercetin tetraglycosides of different Brassica species in µg/g DW.

## Sample Availability

Samples are available from the authors.
