# Supplementary material for: Flavonoid Glycosides in Brassica Species Respond to UV-B Depending on Exposure Time and Adaptation Time
Source: Molecules. 2021 Jan 18;26(2):494. doi: 10.3390/molecules26020494 (PMC7831952; doi:10.3390/molecules26020494)
Supplement: Supplementary file 1 [file molecules-26-00494-s001.pdf]

molecules

MDPI

Supplementary Material

Flavonoid Glycosides in *Brassica* Species Respond to UV-B Dependence on Exposure Time and Adaptation Time

Susanne Neugart [1,*] and Christiane Bumke-Vogt [2]

[1] Division Quality and Sensory of Plant Products, Georg-August-Universität Göttingen, Carl-Sprengel-Weg 1, D-37075 Goettingen, Germany
[2] Leibniz-Institute of Vegetable and Ornamental Crops, Theodor-Echtermeyer-Weg 1, 14979 Grossbeeren, Germany; bumke@igzev.de
* Correspondence: susanne.neugart@uni-goettingen.de; Tel: +49-0551-39-27958

Table S1: Kaempferol diglycosides of different *Brassica* species in µg/g DW. Asterix indicate differences due to the UV-B treatment at the certain time point significant at * $p \leq 0.05$, ** $p \leq 0.01$, *** $p \leq 0.005$
K: kaempferol; soph: sophorose; glc: glucose; cou: coumaroyl; caf: caffeoyl; fer: feruloyl; hfer: hydroxyferuloyl; sin: sinapoyl; h: hour; cond: condition

Brassica			K-3-soph	K-3-cou-soph	K-3-caf-soph	K-3-fer-soph	K-3-hfer-soph	K-3-sin-soph
rapa	Day 1	h	n.d.	n.d.				
		cond			**	**		
		h × cond					*	
	Day 4	h	n.d.	n.d.		*		*
		cond						
		h × cond						
	Day 7	h	n.d.	n.d.			***	*
		cond				***		
		h × cond						
nigra	Day 1	h	n.d.	n.d.	***	n.d.		***
		cond			**		**	

		h × cond			***			
	Day 4	h	n.d.	n.d.	***	n.d.		
		cond						*
		h × cond						
	Day 7	h	n.d.	n.d.		n.d.	*	***
		cond						
		h × cond						
oleracea	Day 1	h	n.d.	n.d.		n.d.		
		cond						
		h × cond						
	Day 4	h	n.d.	n.d.	**	n.d.		*
		cond						
		h × cond						
	Day 7	h	n.d.	n.d.	***	n.d.		
		cond					**	
		h × cond			***			
juncea	Day 1	h	n.d.	n.d.			n.d.	
		cond			*			
		h × cond						*
	Day 4	h	n.d.	n.d.		*	n.d.	
		cond						
		h × cond			*			
	Day 7	h	n.d.	n.d.			n.d.	
		cond						
		h × cond						***
napus	Day 1	h	n.d.	***			n.d.	
		cond						
		h × cond						
	Day 4	h	n.d.				n.d.	
		cond						

		h × cond						
	Day 7	h	n.d.	*	*	***	n.d.	
		cond						
		h × cond						
carinata	Day 1	h	n.d.	n.d.		n.d.		*
		cond					*	***
		h × cond						
	Day 4	h	n.d.	n.d.		n.d.		
		cond			*		*	
		h × cond						
	Day 7	h	n.d.	n.d.		n.d.		
		cond						
		h × cond						***

Table S2: Quercetin diglycosides of different *Brassica* species in µg/g DW. Asterix indicate differences due to the UV-B treatment at the certain time point significant at * $p \leq 0.05$, ** $p \leq 0.01$, *** $p \leq 0.005$

Q: quercetin; soph: sophorose; glc: glucose; cou: coumaroyl; caf: caffeoyl; fer: feruloyl; hfer: hydroxyferuloyl; sin: sinapoyl; h: hour; cond: condition

Brassica			Q-3-soph	Q-3-cou-soph	Q-3-caf-soph	Q-3-fer-soph	Q-3-hfer-soph	Q-3-sin-soph
rapa	Day 1	h	n.d.	n.d.	n.d.	n.d.		n.d.
		cond						
		h × cond						
	Day 4	h	n.d.	n.d.	n.d.	n.d.		n.d.
		cond						
		h × cond						
	Day 7	h	n.d.	n.d.	n.d.	n.d.		n.d.
		cond						
		h × cond						
nigra	Day 1	h	n.d.	n.d.	n.d.	n.d.		n.d.
		cond						
		h × cond						
	Day 4	h	n.d.	n.d.	n.d.	n.d.		n.d.
		cond						
		h × cond						
	Day 7	h	n.d.	n.d.	n.d.	n.d.		n.d.
		cond						
		h × cond						
oleracea	Day 1	h	n.d.	n.d.	n.d.	n.d.	n.d.	
		cond						
		h × cond						
	Day 4	h	n.d.	n.d.	n.d.	n.d.	n.d.	*
		cond						
		h × cond						
	Day 7	h	n.d.	n.d.	n.d.	n.d.	n.d.	

		cond						*
		h × cond						
juncea	Day 1	h	n.d.	n.d.	n.d.	n.d.		
		cond						
		h × cond						
	Day 4	h	n.d.	n.d.	n.d.	n.d.		
		cond						
		h × cond						
	Day 7	h	n.d.	n.d.	n.d.	n.d.		
		cond						
		h × cond						
napus	Day 1	h	n.d.	n.d.	n.d.	n.d.		n.d.
		cond						
		h × cond						
	Day 4	h	n.d.	n.d.	n.d.	n.d.		n.d.
		cond						
		h × cond						
	Day 7	h	n.d.	n.d.	n.d.	n.d.		n.d.
		cond						
		h × cond						
carinata	Day 1	h	n.d.	n.d.	n.d.	n.d.	n.d.	n.d.
		cond						
		h × cond						
	Day 4	h	n.d.	n.d.	n.d.	n.d.	n.d.	n.d.
		cond						
		h × cond						
	Day 7	h	n.d.	n.d.	n.d.	n.d.	n.d.	n.d.
		cond						
		h × cond						

Table S3: Kaempferol triglycosides of different *Brassica* species in µg/g DW. Asterix indicate differences due to the UV-B treatment at the certain time point significant at * $p \leq 0.05$, ** $p \leq 0.01$, *** $p \leq 0.005$

K: kaempferol; soph: sophoroside; glc: glucose; cou: coumaroyl; caf: caffeoyl; fer: feruloyl; hfer: hydroxyferuloyl; sin: sinapoyl; h: hour; cond: condition

Brassica			K-3-soph-7-glc	K-3-cou-soph-7-glc	K-3-caf-soph-7-glc	K-3-fer-soph-7-glc	K-3-hfer-soph-7-glc	K-3-sin-soph-7-glc	K-3-disin-soph-7-glc	K-3-sin,fer-soph-7-glc
rapa	Day 1	h								
		cond								
		h × cond								
	Day 4	h		**			*	**		
		cond			*					
		h × cond								
	Day 7	h								
		cond				*				
		h × cond								
nigra	Day 1	h				*	*		n.d.	n.d.
		cond								
		h × cond								
	Day 4	h		***					n.d.	n.d.
		cond		*						
		h × cond								
	Day 7	h					*		n.d.	n.d.
		cond								
		h × cond								
oleracea	Day 1	h							***	***
		cond								
		h × cond				*				

	Day 4	h	***	***	***	***	***		**	***
		cond	**		*	***	*	***		
		h × cond		**	*	***	***			
	Day 7	h								
		cond		***	**	***	*	***		***
		h × cond	***							
juncea	Day 1	h				*				
		cond		*		*				
		h × cond								
	Day 4	h		*				***		
		cond								
		h × cond								
	Day 7	h						***		
		cond	*	*				*		
		h × cond								
napus	Day 1	h			***		***		n.d.	n.d.
		cond								
		h × cond								
	Day 4	h						***	n.d.	n.d.
		cond			*					
		h × cond							n.d.	n.d.
	Day 7	h								
		cond								
		h × cond								
carinata	Day 1	h	*							
		cond								

	h × cond			*					
Day 4	h		*	***				n.d.	n.d.
	cond	*	*	***					
	h × cond	*	*						
Day 7	h		*		***			n.d.	n.d.
	cond								
	h × cond								

Table S4: Quercetin triglycosides of different *Brassica* species in µg/g DW. Asterix indicate differences due to the UV-B treatment at the certain time point significant at * $p \leq 0.05$, ** $p \leq 0.01$, *** $p \leq 0.005$

Q: quercetin; soph: 9inapoyl9; glc: glucose; cou: coumaroyl; caf: caffeoyl; fer: feruloyl; hfer: hydroxyferuloyl; sin: 9inapoyl; h: hour; cond: condition

Brassica			Q-3-soph-7-glc	Q-3-cou-soph-7-glc	Q-3-caf-soph-7-glc	Q-3-fer-soph-7-glc	Q-3-hfer-soph-7-glc	Q-3-sin-soph-7-glc
rapa	Day 1	h		n.d.		n.d.		
		cond						
		h × cond	*					
	Day 4	h		n.d.		n.d.		**
		cond						
		h × cond	*					
	Day 7	h		n.d.		n.d.		
		cond			*			
		h × cond						
nigra	Day 1	h		n.d.	n.d.	n.d.	***	n.d.
		cond					**	
		h × cond					***	
	Day 4	h		n.d.	n.d.	n.d.		n.d.
		cond	*					
		h × cond						
	Day 7	h		n.d.	n.d.	n.d.		n.d.
		cond						
		h × cond						
oleracea	Day 1	h		n.d.		n.d.	n.d.	***
		cond						
		h × cond						
	Day 4	h	**	n.d.	***	n.d.	n.d.	**
		cond			***			

		h × cond			***			
	Day 7	h		n.d.		n.d.	n.d.	
		cond						***
		h × cond						
juncea	Day 1	h		n.d.	*	n.d.		n.d.
		cond						
		h × cond					*	
	Day 4	h		n.d.		n.d.		n.d.
		cond						
		h × cond						
	Day 7	h		n.d.		n.d.		n.d.
		cond						
		h × cond						
napus	Day 1	h		n.d.	n.d.	n.d.	n.d.	n.d.
		cond						
		h × cond						
	Day 4	h		n.d.	n.d.	n.d.	n.d.	n.d.
		cond						
		h × cond						
	Day 7	h		n.d.	n.d.	n.d.	n.d.	n.d.
		cond						
		h × cond						
carinata	Day 1	h		n.d.		n.d.	***	
		cond					***	
		h × cond					*	
	Day 4	h		n.d.	n.d.	n.d.	n.d.	n.d.
		cond	*					
		h × cond	*					
	Day 7	h		n.d.	n.d.	n.d.	n.d.	n.d.

cond
h × cond

Table S5: Kaempferol tetraglycosides of different *Brassica* species in µg/g DW. Asterix indicate differences due to the UV-B treatment at the certain time point significant at * $p \leq 0.05$, ** $p \leq 0.01$, *** $p \leq 0.005$

K: kaempferol; soph: 12inapoyl12; glc: glucose; cou: coumaroyl; caf: caffeoyl; fer: feruloyl; hfer: hydroxyferuloyl; sin: 12inapoyl; h: hour; cond: condition

Brassica			K-3-soph-7-diglc	K-3-cou-soph-7-diglc	K-3-caf-soph-7-diglc	K-3-fer-soph-7-diglc	K-3-hfer-soph-7-diglc	K-3-sin-soph-7-diglc	K-3-disin-striglc-7-glc
rapa	Day 1	h	n.d.	n.d.	n.d.	n.d.	n.d.	n.d.	n.d.
		cond							
		h × cond							
	Day 4	h	n.d.	n.d.	n.d.	n.d.	n.d.	n.d.	n.d.
		cond							
		h × cond							
	Day 7	h	n.d.	n.d.	n.d.	n.d.	n.d.	n.d.	n.d.
		cond							
		h × cond							
nigra	Day 1	h	n.d.	n.d.	***				n.d.
		cond							
		h × cond					**		
	Day 4	h	n.d.	n.d.	***		***	***	n.d.
		cond							
		h × cond							
	Day 7	h	n.d.	n.d.		*			n.d.
		cond							
		h × cond					*		
oleracea	Day 1	h	n.d.	n.d.	n.d.		**		
		cond							
		h × cond							
	Day 4	h	n.d.	n.d.	n.d.	*	***	*	**

		cond					*		**
		h × cond							
	Day 7	h	n.d.	n.d.	n.d.		**		*
		cond				***	***	***	*
		h × cond							
juncea	Day 1	h	n.d.	n.d.	n.d.		n.d.		n.d.
		cond							
		h × cond							
	Day 4	h	n.d.	n.d.	n.d.		n.d.		n.d.
		cond							
		h × cond							
	Day 7	h	n.d.	n.d.	n.d.		n.d.		n.d.
		cond							
		h × cond							
napus	Day 1	h	n.d.	n.d.	n.d.	***	*		
		cond				*			
		h × cond				*			
	Day 4	h	n.d.	n.d.	n.d.	n.d.	***		
		cond							
		h × cond							
	Day 7	h	n.d.	n.d.		n.d.			
		cond							
		h × cond			*				*
carinata	Day 1	h	n.d.	n.d.	n.d.				
		cond							
		h × cond							
	Day 4	h	n.d.	n.d.	n.d.				
		cond							
		h × cond							

Day 7	h	n.d.	n.d.	n.d.				***
	cond						*	**
	h × cond							**

Table S6: Quercetin tetraglycosides of different *Brassica* species in µg/g DW. Asterix indicate differences due to the UV-B treatment at the certain time point significant at * $p \leq 0.05$, ** $p \leq 0.01$, *** $p \leq 0.005$

Q: quercetin; soph: 15inapoyl15; glc: glucose; cou: coumaroyl; caf: caffeoyl; fer: feruloyl; hfer: hydroxyferuloyl; sin: 15inapoyl; h: hour; cond: condition

Brassica			Q-3-soph-7-diglc	Q-3-cou-soph-7-diglc	Q-3-caf-soph-7-diglc	Q-3-fer-soph-7-diglc	Q-3-hfer-soph-7-diglc	Q-3-sin-soph-7-diglc	Q-3-disin-triglc-7-glc
rapa	Day 1	h	n.d.	n.d.	n.d.	n.d.	n.d.	n.d.	n.d.
		cond							
		h × cond							
	Day 4	h	n.d.	n.d.	n.d.	n.d.	n.d.	n.d.	n.d.
		cond							
		h × cond							
	Day 7	h	n.d.	n.d.	n.d.	n.d.	n.d.	n.d.	n.d.
		cond							
		h × cond							
nigra	Day 1	h	n.d.	n.d.	n.d.	n.d.	n.d.	n.d.	n.d.
		cond							
		h × cond							
	Day 4	h	n.d.	n.d.	n.d.	n.d.	n.d.	n.d.	n.d.
		cond							
		h × cond							
	Day 7	h	n.d.	n.d.	n.d.	n.d.	n.d.	n.d.	n.d.
		cond							
		h × cond							
oleracea	Day 1	h	n.d.	n.d.	n.d.	n.d.	n.d.		*
		cond							
		h × cond							
	Day 4	h	n.d.	n.d.	n.d.	n.d.	n.d.		*

		cond						***	
		h × cond							
	Day 7	h	n.d.	n.d.	n.d.	n.d.	n.d.		
		cond						***	
		h × cond							
juncea	Day 1	h	n.d.	n.d.	n.d.	n.d.	n.d.	n.d.	n.d.
		cond							
		h × cond							
	Day 4	h	n.d.	n.d.	n.d.	n.d.	n.d.	n.d.	n.d.
		cond							
		h × cond							
	Day 7	h	n.d.	n.d.	n.d.	n.d.	n.d.	n.d.	n.d.
		cond							
		h × cond							
napus	Day 1	h	n.d.	n.d.	n.d.	n.d.	n.d.	n.d.	
		cond							
		h × cond							
	Day 4	h	n.d.	n.d.	n.d.	n.d.	n.d.	n.d.	
		cond							
		h × cond							*
	Day 7	h	n.d.	n.d.	n.d.	n.d.	n.d.	n.d.	
		cond							
		h × cond							*
carinata	Day 1	h	n.d.	n.d.	n.d.	n.d.	n.d.	n.d.	
		cond							
		h × cond							
	Day 4	h	n.d.	n.d.	n.d.	n.d.	n.d.	n.d.	
		cond							
		h × cond							*

Day 7	h	n.d.	n.d.	n.d.	n.d.	n.d.	n.d.	
	cond							
	h × cond							***

Table S7: Quercetin tetraglycosides of different *Brassica* species in µg/g DW. Asterix indicate differences due to the UV-B treatment at the certain time point significant at * $p \leq 0.05$, ** $p \leq 0.01$, *** $p \leq 0.005$

Q: quercetin; soph: sinapoyl; glc: glucose; cou: coumaroyl; caf: caffeoyl; fer: feruloyl; hfer: hydroxyferuloyl; sin: sinapoyl; h: hour; cond: condition

Brassica			Chl	Cou-mal	Caf-mal	Fer-mal	Hfer-mal	Sin-mal	Disin-gent	Sin,fer-gent	Trisin-gent	Disin,fer-gent
rapa	Day 1	h										
		cond										
		h × cond										
	Day 4	h			*			***		*		
		cond	*				**					
		h × cond										
	Day 7	h										n.d.
		cond	*				*					
		h × cond										
nigra	Day 1	h	***	n.d.	n.d.	n.d.	n.d.	n.d.				
		cond	*									
		h × cond										
	Day 4	h		n.d.	n.d.	n.d.	n.d.	n.d.	*			
		cond										
		h × cond										
	Day 7	h		n.d.	n.d.	n.d.	n.d.	n.d.				
		cond							*			
		h × cond										*

oleracea	Day 1	h		n.d.	n.d.	n.d.	n.d.	n.d.	*	*	*	*
		cond										
		h × cond										
	Day 4	h		n.d.	n.d.	n.d.	n.d.	n.d.	***	***		***
		cond									*	***
		h × cond										***
	Day 7	h		n.d.	n.d.	n.d.	n.d.	n.d.				
		cond									*	
		h × cond										
juncea	Day 1	h			*					*		
		cond										
		h × cond								*		
	Day 4	h										
		cond		***		**		*				
		h × cond										
	Day 7	h	*		***		***	***				n.d.
		cond										
		h × cond										
napus	Day 1	h		*	*	***			*			
		cond										
		h × cond										
	Day 4	h		*		**	***	***			*	
		cond		**	*	*		***				

		h × cond										
	Day 7	h										
		cond										
		h × cond										
carinata	Day 1	h		n.d.	n.d.	n.d.	n.d.	n.d.	n.d.	n.d.	n.d.	n.d.
		cond										
		h × cond										
	Day 4	h		n.d.	n.d.	n.d.	n.d.	n.d.	n.d.	n.d.	n.d.	n.d.
		cond										
		h × cond										
	Day 7	h	***	n.d.	n.d.	n.d.	n.d.	n.d.	n.d.	n.d.	n.d.	n.d.
		cond	***									
		h × cond										
